# Effects of Early Life Adversity on Tooth Enamel Formation

**DOI:** 10.3389/fdmed.2022.894753

**Published:** 2022-06-15

**Authors:** Ida C. Shaffer, Yukiko Nakano, Aidan Pham, Annabel Short, Antonio Nanci, Yan Zhang, Rozana Shemirani, Pamela K. Den Besten

**Affiliations:** 1 Department of Orofacial Sciences, University of California, San Francisco, San Francisco, CA, United States; 2 Departments of Pediatrics, Anatomy/Neurobiology, Neurology, University of California, Irvine, Irvine, CA, United States; 3 Department of Stomatology, Faculty of Dental Medicine, Université de Montréal, Montreal, QC, Canada

**Keywords:** enamel, early life adversity (ELA), mineralization, ameloblasts, RNAseq, limited bedding and nesting (LBN)

## Abstract

In a systemic effort to survive environmental stress, organ systems fluctuate and adapt to overcome external pressures. The evolutionary drive back toward homeostasis makes it difficult to determine if an organism experienced a toxic exposure to stress, especially in early prenatal and neonatal periods of development. Previous studies indicate that primary human teeth may provide historical records of experiences related to stressors during that early time window. To assess the molecular effects of early life adversity on enamel formation, we used a limited bedding and nesting (LBN) mouse model of early life adversity (ELA) to assess changes in the enamel organ gene expression and enamel matrix mineralization. On average, postnatal day 12 (P12) ELA mice weighed significantly less than the controls. When adjusted for animal weight, ELA molar enamel volume was reduced as compared with the controls, and the relative mineral density of molar enamel was significantly increased. There were no obvious changes in enamel matrix crystal morphology or structure in ELA as compared with the control mouse enamel. RNAseq showed extracellular matrix organization to be the most significantly affected GO and reactome pathways, whereas butanote metabolism was the most significantly altered KEGG pathway. Transcripts expressing the enamel matrix proteins amelogenin (*Amelx*) and enamelin (*Enam*) were among the top 4 most differentially expressed genes. When evaluating molecular mechanisms for the changes in gene expression in ELA enamel organs, we found significantly increased expression of *Dlx3*, while transcripts for clock genes *Per1* and *Nrd1* were downregulated. These findings support the possibility that the developing enamel organ is sensitive to the pressures of early life adversity and produces molecular and structural biomarkers reflecting these challenges.

## INTRODUCTION

The exposome, which includes environmental stress affecting the hypothalamus–pituitary–adrenal (HPA) axis, affects gene transcription and organ phenotype (1, 2). Evidence from our work in characterizing phenotypes of human primary teeth suggests that exposure to early life adversity alters the tooth phenotype (3–5). Adverse early life experiences can affect physiology, including immune function, cognitive, and emotional development, and can influence the risk of developing stress-related psychopathology (6, 7). Therefore, biomarkers, such as those reflected in altered phenotypes in tooth enamel, could be useful screening tools for identifying children at risk of early life stress-related diseases. However, further studies are needed to understand the mechanisms by which alterations in the HPA axis, such as those resulting from early life adversity, affect tooth formation.

To study how early life adversity (ELA) affects tooth enamel formation, we used the limited bedding and nesting mouse model (LBN) developed in the Baram lab (8–10). In this model, pup stress is evoked *via* fragmented maternal care, generated by reducing the amount of nesting material available to the dam beginning at P2 through the end of the study period at P12.

In mice, the incisor continuously erupts so that all stages of enamel development are present over the length of the incisor, while molars are rooted teeth that develop through sequential stages of development similar to human teeth. In molars, enamel formation begins at P2 with the secretion of enamel matrix proteins, including amelogenin, enamelin, and ameloblastin. Following the secretion of matrix proteins, the protein matrix is hydrolyzed first by MMP-20, followed by further hydrolysis with KLK4 in the maturation stage. As the protein is removed, it is replaced by minerals to form the highly mineralized mature enamel matrix. In mouse molars, the maturation stage begins from about P8 and is complete by P15 (11). The coincident timing of mouse molar tooth enamel matrix protein secretion and maturation to the timing of early life stress in the LBN model for ELA makes this ELA model ideal to assess the effects of stress of early life on tooth enamel formation.

## METHODS

### Limited Bedding and Nesting Early Life Adversity (ELA) Mouse Protocol

#### Animals

Dams were Crh-IRES-Cre +/+ (12), and they were paired with Ai14 tdTomato (13) males, both on a C57Bl6 background. The resulting offsprings were Crh-IRES-Cre, Ai14 tdTomato, as previously described (14). Animals were housed in a 12-h light cycle and provided *ad libitum* food and water. All experiments were carried out in accordance with the University of California, Irvine Institutional Animal Care and Use Committee at the University of California-Irvine and were consistent with Federal guidelines.

Early life adversity was imposed on neonatal mice using simulated poverty by limiting nesting and bedding materials in cages between P2 and P12 (9, 10). For the ELA group, a plastic-coated mesh platform was placed ∼2.5 cm above the floor of a standard cage. Cobb bedding was reduced to cover the cage floor sparsely, and one-half of a single nestlet was provided for nesting material on the platform. Control dams and litters resided in standard cages containing ample cobb bedding and one whole nestlet for nesting. Control and experimental cages were undisturbed during P2–P12, housed in temperature-controlled rooms (22°C).

### MicroCT Imaging and Analysis of P12 Mouse Mandibles

#### Mandible Collection

On postnatal day 12 (P12), mandibles were collected from 3 separate litters of control (*N* = 9) and 3 separate litters of ELA (*N* = 10) mice. The mandibles were fixed in 4% PFA for 24 h, and a total of 5 male and 4 female control and 6 male and 4 female ELA mice were selected for microCT imaging.

#### MicroCT Scanning

One hemimandible from each mouse was scanned by micro-computed tomography (microCT) using a Scanco Medical µCT50 at the UCSF Core Center of Muscoloskeletal Biology and Medicine under the Skeletal Biology core. Specimens were scanned at 10.0 µm resolution with 500 ms integration time within a field of view of 15.2 mm (energy parameters of 55 kVP, 109 µA, 6 W, 0.5 mm AI filter). Reconstructions were generated using Scanco Medical’s integrated µCT Evaluation Program V6.5–3 and converted into DICOM files for post-process analysis.

#### Image Analysis

DICOM files were uploaded into Amira software (ThermoFisher, Version 2020.3.1). A non-local means filter [spatial StdDev = 5, intensity StdDev = 0.2, search window [px] = 9, local neighborhood [px] = 3] and an unsharp masking [interpretation = 3D, edge size [px] = 6, edge contrast = 0.5, brightness threshold = 0] image processing filter was applied to increase the contrast between the mineralized enamel matrix and the surrounding dentin. An enamel masking threshold of 8,500–18,000 Hounsfield units (HU) was applied to the segment mineralizing enamel matrix. Relative enamel mineral density was calculated by averaging the greyscale values in Hounsfield units (HU) of all the voxels within this segment. A 3D volume smoothing (px size = 3) was applied to exclude partial volume components. The volume of the mineralizing matrix was calculated by simple voxel counting of the labeled enamel material. Relative enamel density and volume of all the samples were compared relative to body weight by student *t*-tests.

### Enamel Matrix Crystal Structure

To examine the enamel matrix structure, hemimandibles were fixed by immersion in 4% PFA for 24 h and then stored in PBS at 4°C. They were then post-fixed with a 50:50 mixture of 1.5% aqueous potassium ferrocyanide and 1% aqueous osmium tetroxide, dehydrated in a 30–100% graded ethanol series, and processed for embedding in LR White resin (Electron Microscopy Sciences). The polymerized resin blocks were sectioned perpendicular to the hemimandible with an IsoMet low-speed saw (Buehler, Lake Bluff, Il) and then polished using a polisher PowerPro 5000 (Buehler). The samples were imaged using a Hitachi Regulus 8220 scanning electron microscope (SEM) operated at 1 kV using the low angle backscattered detector (LA-BSE) for imaging.

### RNAseq Pathway Analysis

Mandibles of 4 P12 male controls (bodyweight = 5.9 ± 0.4 gm) and 5 P12 male ELA (bodyweight = 5.30 ± 0.4 g) mouse pups were placed in an RNAlater stabilization solution (Invitrogen) and then transferred to phosphate-buffered saline. Enamel organs were removed from the first molars, and mRNA was extracted and purified using a Direct-zol RNA MiniPrep kit (Zymo Research). RNA quality and quantity were assessed using a NanoDrop spectrophotometer and sent to Novogene Corporation Inc. (Sacramento, CA) for RNA sequencing and analysis.

Gene expression was quantified, normalized, and the differential gene expression was assessed using DESeq2 at a *p*-value of < 0.05 (15), followed by an assessment of FDR values (false discovery rate) (16). Pathway enrichment analyses included gene ontology (GO), KEGG, which integrates genomic, chemical, and systemic functional information (17), and reactome pathway analyses.

### qPCR Amplification

P12 enamel organs from control male mice from 2 separate control litters (*N* = 4) and male mice from 2 separate ELA litters (*N* = 5) were collected for qPCR transcript analysis. The expressions of enamel matrix genes *Amelx*, *Enam*, maturation stage enamel matrix proteinase *Klk4,* clock genes *Nr1d1*, and *Per2* (18, 19), *Dlx3* (20), *Igfbp2*, *Igfbp3* (21); as well as *Hsd11b2* were amplified from ELA and control enamel organs following conversion of mRNA to cDNA using SuperScript IV VILO Master Mix (Invitrogen). Relative mRNA expression was quantified by qPCR with PowerUp SYBR Green Master Mix (Applied Biosystems) using primer sets generated by Elim Biopharmaceuticals, Hayward, CA with *Rpl19* used as the reference gene (Table 1). The relative expression of target genes was analyzed using the ∆∆Ct method (22). Significant differences in expression were determined by an independent student *t*-test using fold-change levels.

## RESULTS

### Animal Weight Was Associated With the Density and Volume of the Mineralizing Enamel Matrix

**FIGURE 1 |:**
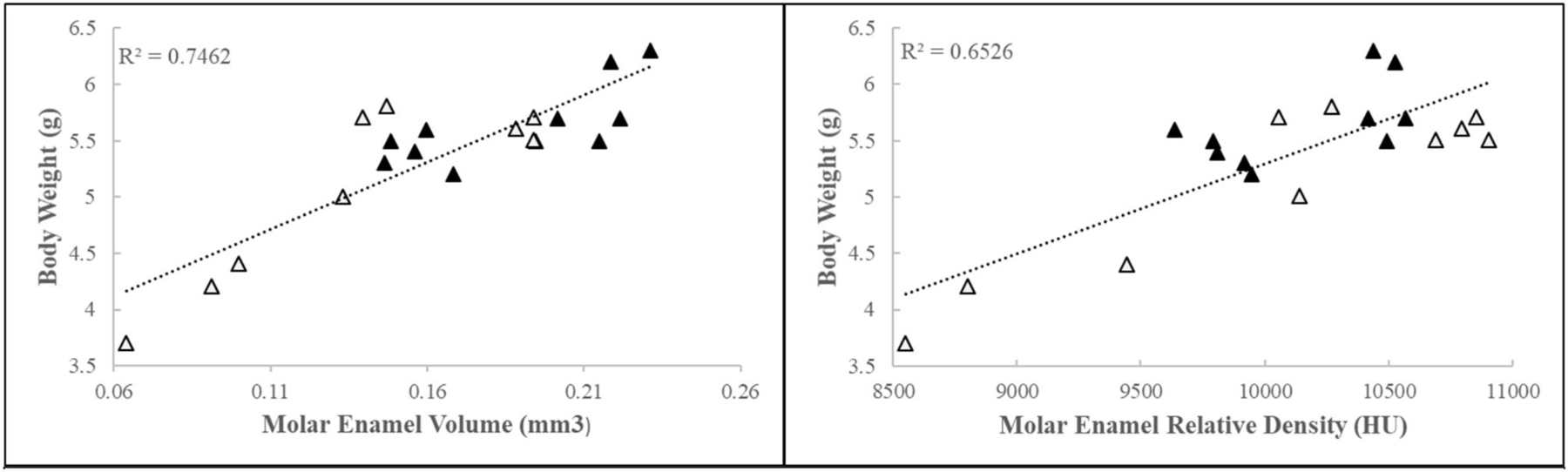
First molar relative enamel mineral density and enamel volume is associated with animal weight. Black triangles = control, white triangles = ELA.

Early life adversity mice collected for further dissection and analyses (4.97 ± 0.8 g, *N* = 18; 6 l) weighed on average 20% less than their control counterparts (6.0 ± 0.5 g, *N* = 26; 7 l). Density, as measured by relative intensity, and volume of the mineralized segment of the enamel matrix were negatively correlated with body weight (Figure 1). These data show that body weight influences mineralized enamel volume and density.

**FIGURE 2 |:**
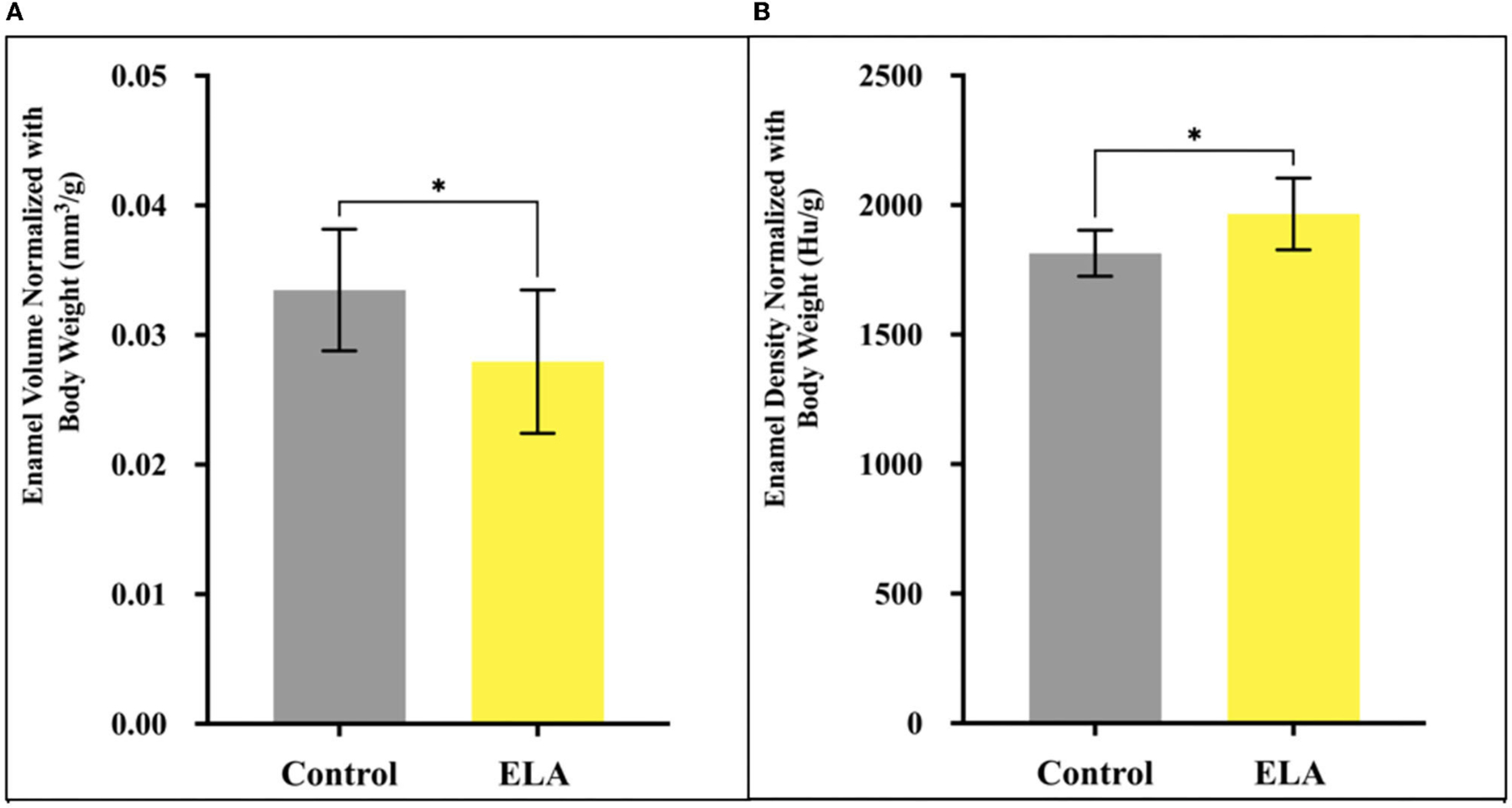
Mineralized enamel volume and relative density in molars from P12 mice, normalized for body weight. **(A)** Enamel volume was significantly less in ELA (n = 10) as compared to controls (n = 10). **(B)** Enamel density was significantly higher in ELA mice as compared to controls *p < 0.05. Error bars: SD.

When we adjusted for body weight, we found that normalized enamel volume and density were similar in male and female mice (data not shown). However, the relative volume of mineralized enamel in ELA was significantly less than that of the controls, and relative density of ELA enamel was significantly increased (Figure 2). Given the large effect of body weight, we checked for normal distribution of weights within the groups using the Shapiro–Wilk test, and while the CTL group passed normality (W=0.89, p=0.21), the ELA mice did not (W=0.83, p=0.04). After excluding the lowest weight values below 4.5 gm, the ELA mice passed the normality test (W=0.83, p=0.075). Therefore, to minimize the effects of weight, these lowest-weight ELA mice were excluded from the remaining analyses.

### SEM Imaging Revealed Normal Enamel Morphology and Crystal Structure

**FIGURE 3 |:**
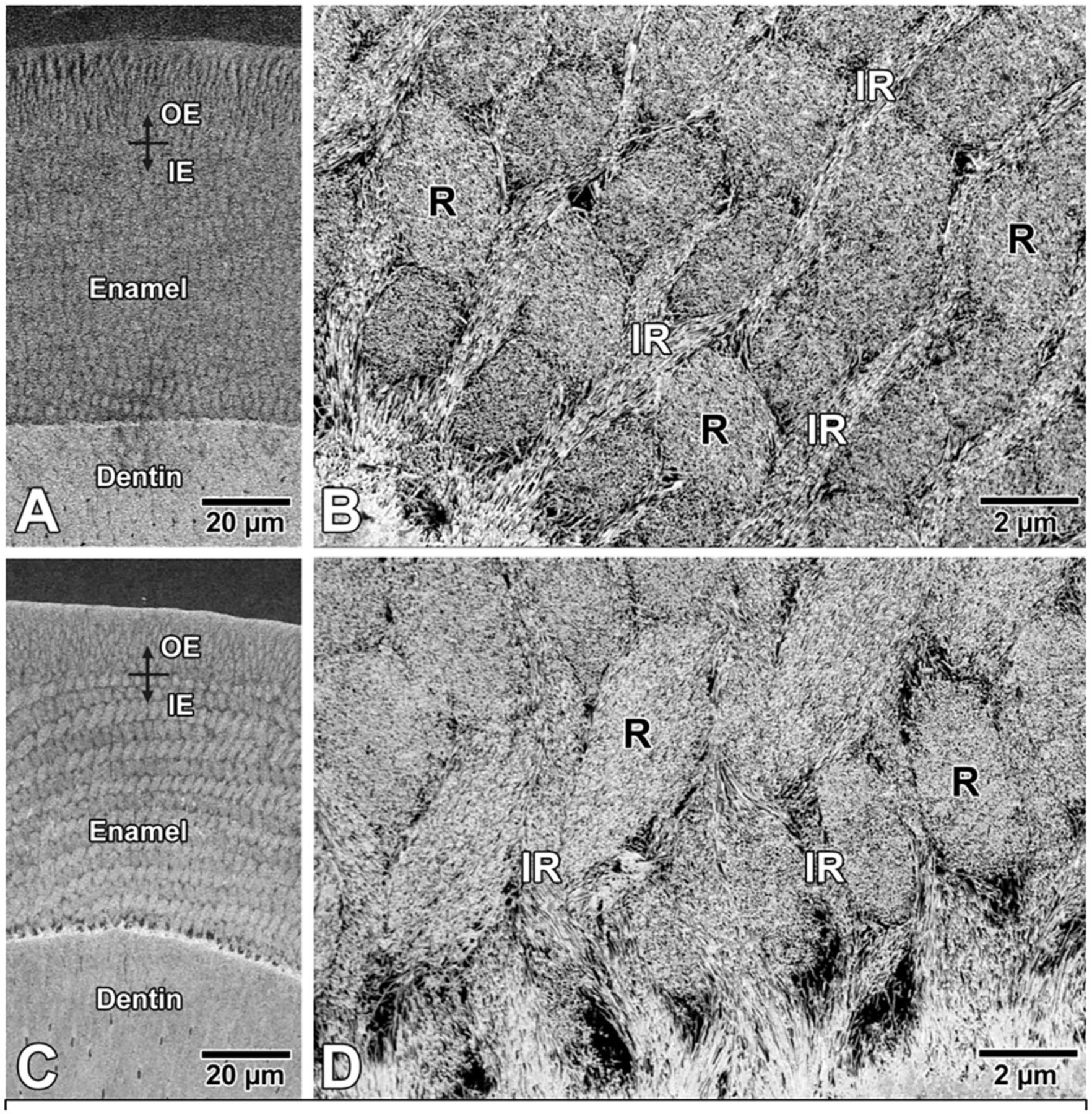
Backscattered electron SEM images of enamel in **(A,B)** control and **(C,D)** ELA mice. Qualitatively, there are no differences in the structural organization of rod (R)-interrod (IR) enamel, and **(B,D)** also no apparent difference in appearance of crystal profiles. IE, inner enamel; OE, outer enamel.

Backscattered SEM images of enamel from P12 control mice (Figures 3A,B) and ELA mice (Figures 3C,D), both showed no difference in the structural organization of the enamel layer and appearance of the enamel crystals.

### RNAseq Seq Analysis Showed Significant Differences in Gene Expression in Enamel Organs From ELA as Compared With Control Mice

**FIGURE 4 |:**
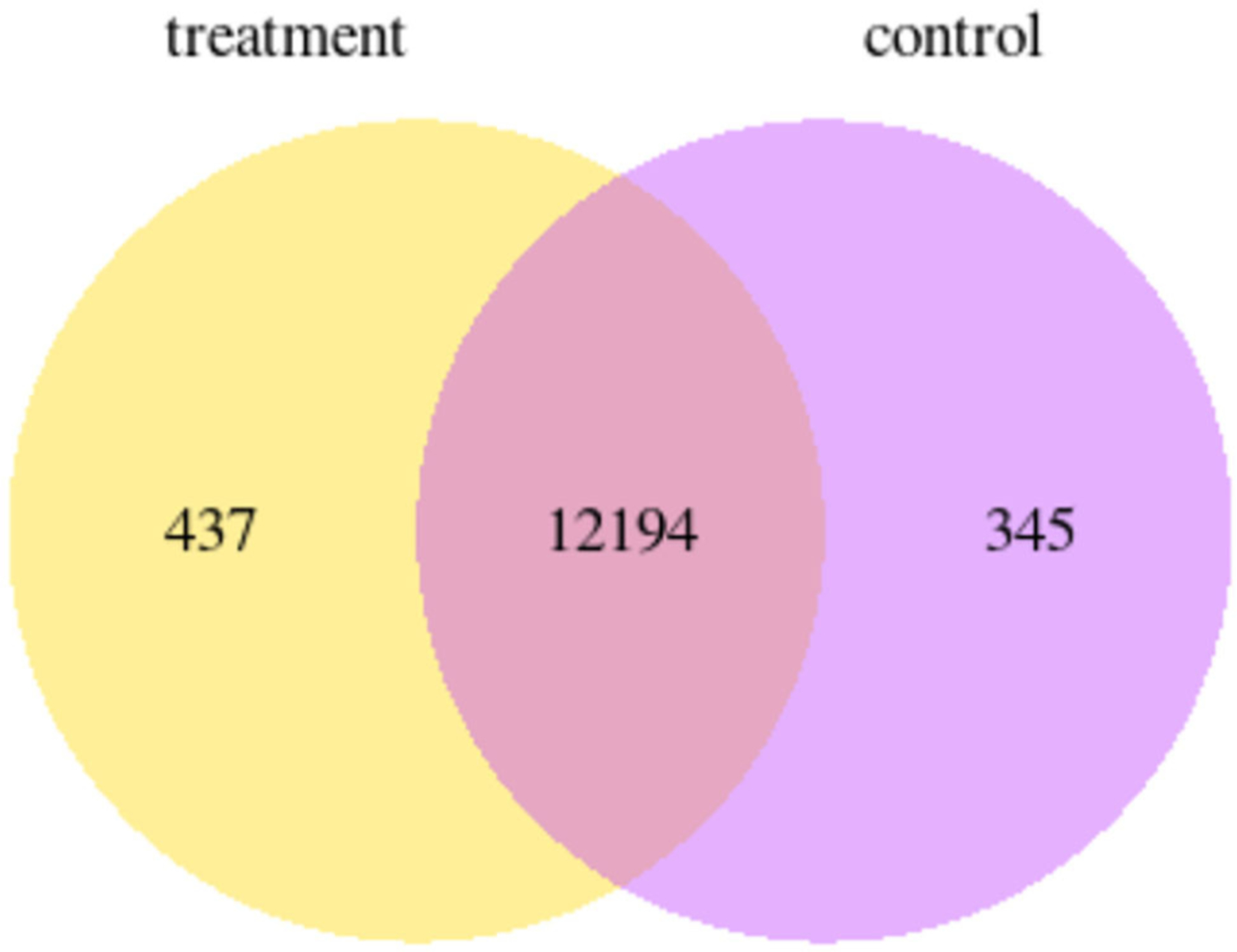
Venn diagram showing the number of genes that are uniquely expressed within each group yellow = ELA mice, purple = weight-matched controls.

RNAseq of P12 enamel organs from control and ELA mice showed 437 genes uniquely expressed in ELA and 345 genes uniquely expressed in control enamel organs (GEO#GSE199982; Figure 4).

There were 81 differentially expressed genes (DEGs) upregulated in ELA p12 mouse’s first molar enamel organs and 62 downregulated genes as compared with the controls (p adjusted <0.05). Secretory enamel matrix proteins, including amelogenin, enamelin, and ameloblastin, were among the most highly upregulated genes in ELA mice. However, there were no differences in the relative expression of the maturation stage matrix proteins odam and amelotin. Genes for metalloproteinases, including MMP20, found primarily in the secretory stage, and KLK4, found primarily in the maturation stage, were also not differentially expressed in ELA as compared with control enamel organs.

Go pathway analysis showed extracellular matrix organization and structure as the most significantly ELA-altered pathways. KEGG pathways that were most significantly altered by ELA were butanoate metabolism, and synthesis and degradation of ketone bodies. The most significantly altered reactome pathway was extracellular matrix organization (see Table 2 and Supplementary Figures 1–3).

### qPCR Analysis Showed Significant Differences in the Expression of Genes Associated With Amelogenin Expression in ELA and Control P12 Molars

**FIGURE 5 |:**
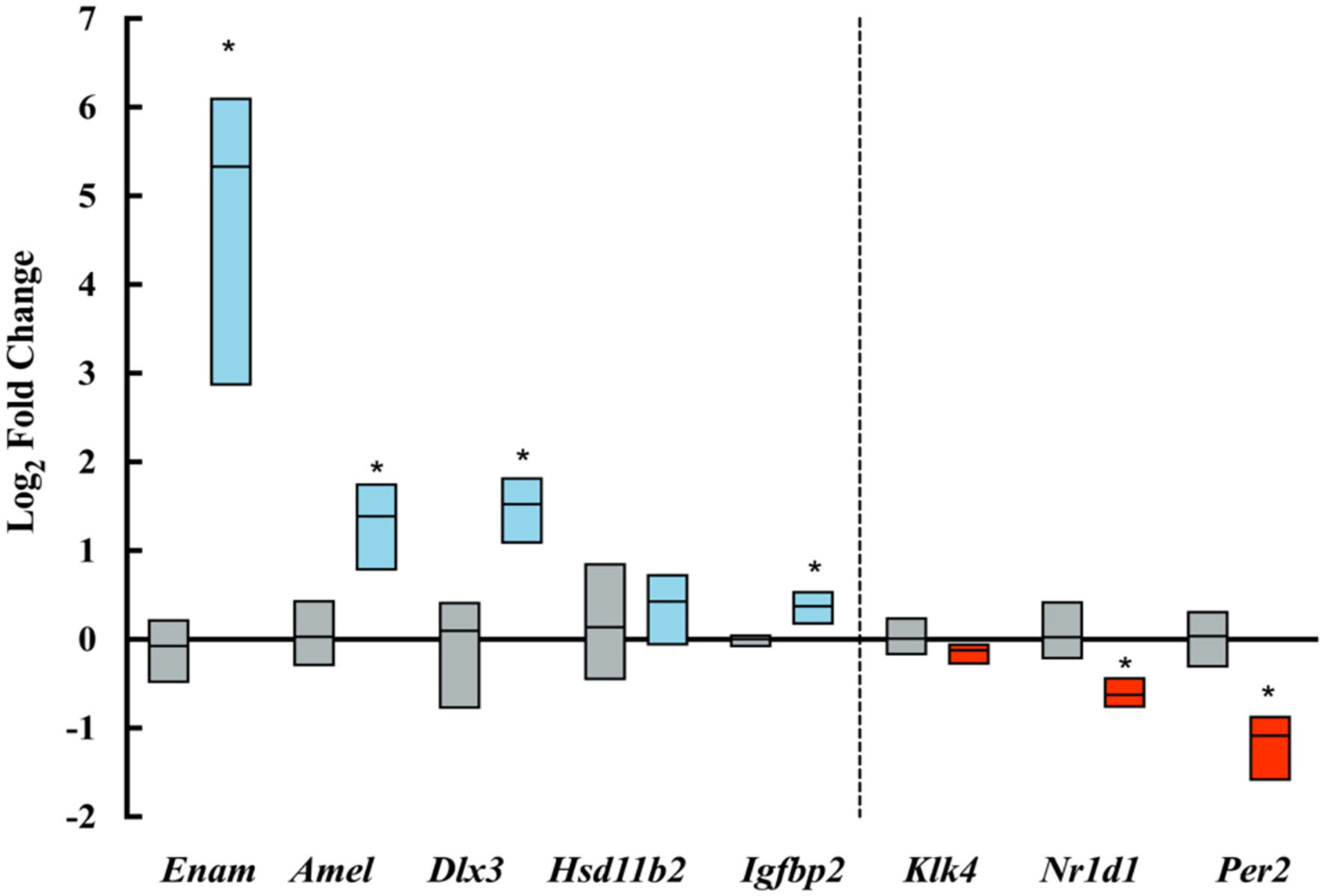
Log-fold changes of transcripts amplified by qPCR. Blue boxes show upregulated transcripts and red boxes show downregulated transcripts expressed in ELA enamel organs as compared to controls (gray boxes). *p <= 0.05.

PCR amplification and analysis showed a significant increase in the expression of amelogenin and enamelin in ELA as compared with control mice. Clock genes *Nr1d1* and *Per2*, which are associated with increased amelogenin expression (18, 19) were, however, downregulated in ELA enamel organs. Expression of *Igfb2,* which is associated with IGF-related amelogenin expression (21) was upregulated, and *Dlx3,* a transcription factor associated with amelogenin expression (20) was significantly increased. There were no differences in the expression of *Klk4* and *Hsd11b2* gene expression (Figure 5).

## DISCUSSION

Our results show that in mice, the experience of early life adversity alters tooth enamel formation. Overall, we found a negative association between body weight and the relative density and volume of the mineralized enamel. This indicates that factors that influence overall growth, as reflected in body weight, also influence enamel mineralization. The bodyweight of ELA mice is negatively associated with plasma corticosterone levels (23), and intracellular corticosterone concentration can be regulated by corticosteroid 11-beta-dehydrogenase isozyme 2, expressed by *Hsd11b2.* However, we found low expression of Hsd11b2 in the enamel organ, and PCR amplification showed no significant differences in *Hsd11b2* expression in ELA as compared with weight-matched controls. This suggests that cellular corticosterone in the enamel organ does not have a major role in altering enamel matrix mineralization, but rather, in ELA mice, these changes are associated with systemic effects resulting from the dysregulation of the hypothalamus pituitary adrenal axis (HPA).

A cellular effect of ELA that may affect amelogenesis is suggested by the enriched KEGG pathway, butyrate metabolism. Butyrate metabolism describes the metabolic fate of short-chain fatty acids or short-chain alcohols that are typically produced by intestinal fermentation. Our findings of alterations in the KEGG butyrate metabolism pathway in enamel organs of ELA mice are consistent with studies that have shown LBN models of ELA rats to have increased intestinal permeability, decreased microbial alpha diversity, and reduced butyrate-producing microbes (24). Butyrate metabolism is associated with the production of ketone bodies (25), a pathway that was also affected in ELA enamel organs.

What was unexpected to us was the significant increase in relative density of the mineralized enamel matrix in the weight normalized ELA mice as compared with controls. This relative increase in mineral density in ELA mouse enamel is consistent with our findings in human primary mandibular incisors. Enamel density in primary mandibular incisors is positively associated with internalizing symptoms (5), and internalizing symptoms in humans are associated with early life adversity (26). These studies of ELA mice therefore support the possibility that early life adversity, related to disrupted maternal care (23), can be reflected in the increased enamel mineralization. SEM analysis did not show obvious differences in the structure of enamel crystals in the mineralizing enamel matrix of ELA molars as compared to controls, though qualitative SEM studies to assess possible changes related to the timing of enamel mineralization will require additional studies.

We found that mRNA transcript for the enamel matrix proteins, amelogenin and enamelin, were highly upregulated in ELA enamel organs as compared with the controls. To explore possible mechanisms by which ELA alters amelogenin expression, we evaluated several candidate pathways. C/EBP*α*, a transcription factor for amelogenin in ELA and controls showed no evidence of differential expression by RNAseq (data not shown). Clock genes, *Nr1d1* and *Per2,* which have been positively associated with amelogenin expression (18) were downregulated in ELA mice. Thrombospondin 2, a member of the multifunctional family of glycoproteins, has been shown to increase amelogenin expression (27); however our RNA seq data showed downregulation of thrombospondin 2 in ELA mouse enamel organs. Consistent with previous studies showing that insulin-like growth factor binding proteins (*Igfbp2*, and *Igfbp3*) can regulate the expression pattern of both *Amelx* (21) and *Enam* (28), RNAseq and qPCR data showed a small but significant upregulation in *Igfbp2* in ELA as compared with the control enamel organ.

*Dlx3,* which *in vitro* has been shown to upregulate expression of the enamel matrix protein genes *Amelx*, *Enam*, *Klk4*, and *Odam* (29), was the most highly upregulated of the candidate genes. However, while our RNAseq analysis showed the upregulation of amelogenin and enamelin expression in ELA enamel organs, we found no changes in *KLK4* and *Odam* expression. Duverger et al. (30) reported that *in vivo* DLX3 loss of function has no effect on the expression of the major enamel matrix proteins (including *Amelx* and *Enam*) and proteinases (including KLK4), however, the expression of ion transporters and carbonic anhydrase are affected. Our RNAseq analysis showed significant upregulation of carbonic anhydrase transcripts *Car6*, *Car3*, and *Car12*, which in maturation stage enamel, function to synthesize bicarbonate, which is then transported to the mineralizing enamel matrix to neutralize protons produced by the formation of hydroxyapatite (31, 32). This neutralization of the mineralizing enamel matrix then allows the continued growth of hydroxyapatite crystals. It may be that the relative increase in enamel mineral in weight-matched ELA mice is related to a Dlx3-mediated increase in bicarbonate synthesis to allow more rapid growth of hydroxyapatite crystals.

In the brain, *Dlx* isoforms induce the synthesis of glutamic acid decarboxylase (GAD1 and GAD2) (33), which increases GABA synthesis. Though *Dlx3* has not been associated with the regulation of GABA synthesis, RNA seq data showed upregulated genes for enzymes involved in the breakdown of GABA (*Abat* and *Aldh5A1*) in ELA mice, suggesting the possibility that GABA related pathways are involved in changes in gene expression in enamel organs of the ELA mouse model. *Dlx3* transcription is mediated through Wnt signaling, which has a critical role in amelogenesis (34) and hippocampal neurogenesis (35). These findings suggest the importance of further studies to characterize the effects of early life adversity on Wnt signaling and its effects on enamel maturation.

Taken together our findings show the dysregulation of multiple genes in the enamel organ of mice exposed to early life adversity through limited bedding and nesting and disrupted maternal behaviors. It is not clear why amelogenin and enamelin expression are upregulated in enamel organs from ELA mice, or how increased expression of these genes influences enamel matrix mineralization relative to body weight in the ELA mouse model. However, enhanced enamel mineralization relative to body weight in ELA mice may be specifically associated with increased *Dlx3* expression to drive the upregulation of carbonic anhydrase synthesis, resulting in more rapid mineralization of hydroxyapatite crystals.

Increased relative mineral density in molars of mice exposed to early life adversity is also consistent with the association of early life adversity with accelerated biological aging (36, 37). Therefore, it may be that the enamel from human primary teeth, which mineralize in early life, can provide biomarkers to identify individuals with risk factors associated with early life stress. Taken together, these studies support the concept that the developing tooth enamel organ is a useful model to explore cellular mechanisms related to changes in the HPA axis during development.

## Supplementary Material

Supplemental pdf

## Figures and Tables

**TABLE 1 |:** qPCR primer sequences.

Gene name	NCBI gene ID	Region	Primer sequence (5′ -> 3′)
Amelx	11704	4–24	Fwd: GGGACCTGGATTTTGTTTGCC
		119–99	Rev: TTCAAAGGGGTAAGCACCTCA
Enam	13801	565–583	Fwd: GGACGGCCAAAGTTCAGCA
		734–716	Rev: GGTGGGTCATCTGGAGGTG
Klk4	56640	236–254	Fwd: CGGGAGTCTTGGTGCATCC
		337–316	Rev: CTTGGGAGCCTTTCAGGTTATG
Dlx3	1747	555–573	Fwd: CCGAGGTTCGCATGGTGAA
		672–652	Rev: AAGGCCAGATACTGGGCTTTC
Igfbp2	16008	371–390	Fwd: CAGACGCTACGCTGCTATCC
		510–490	Rev: CCCTCAGAGTGGTCGTCATCA
Igfbp3	16009	225–2246	Fwd: TCTAAGCGGGAGACAGAATACG
		2315–2295	Rev: CTCTGGGACTCAGCACATTGA
Nr1d1	217166	1002–1021	Fwd: TTTTTCGCCGGAGCATCCAA
		1197–1178	Rev: ATCTCGGCAAGCATCCGTTG
Per2	8864	922–941	Fwd: CTTGATGCTCGCCATCCACA
		1069–1050	Rev: TATCTTCCTGCTCCACGGGT
Hsd11b2	15484	384–404	Fwd: GGTTGTGACACTGGTTTTGGC
		565–545	Rev: AGAACACGGCTGATGTCCTCT
Rpl19	19921	467–488	Fwd: ATGAGTATGCTCAGGCTACAGA
		570–550	Rev: GCATTGGCGATTTCATTGGTC

**TABLE 2 |:** Top pathways in enamel organs alter by early-life adversity.

GO	KEGG	Reactome
Extracellular matrix organization	Butanoate metabolism	Extracellular matrix organization
Proteinaceous extracellular matrix	Synthesis and degradation of ketone bodies	Degradation of the extracellular matrix
Extracellular matrix components	Malaria	Integrin cell surface interactions

## Data Availability

The datasets presented in this study can be found in online repositories. The names of the repository/repositories and accession number(s) can be found at: https://www.ncbi.nlm.nih.gov/, GSE199982.
