# Supplementary material for: Effects of Early Life Adversity on Tooth Enamel Formation
Source: Front Dent Med. Author manuscript; Available in PMC 2023 Apr 6. (PMC10079274; doi:10.3389/fdmed.2022.894753)
Supplement: Supplemental pdf [file NIHMS1829497-supplement-Supplemental_pdf.pdf]

Supplementary Figures:

RNAseq pathway analysis

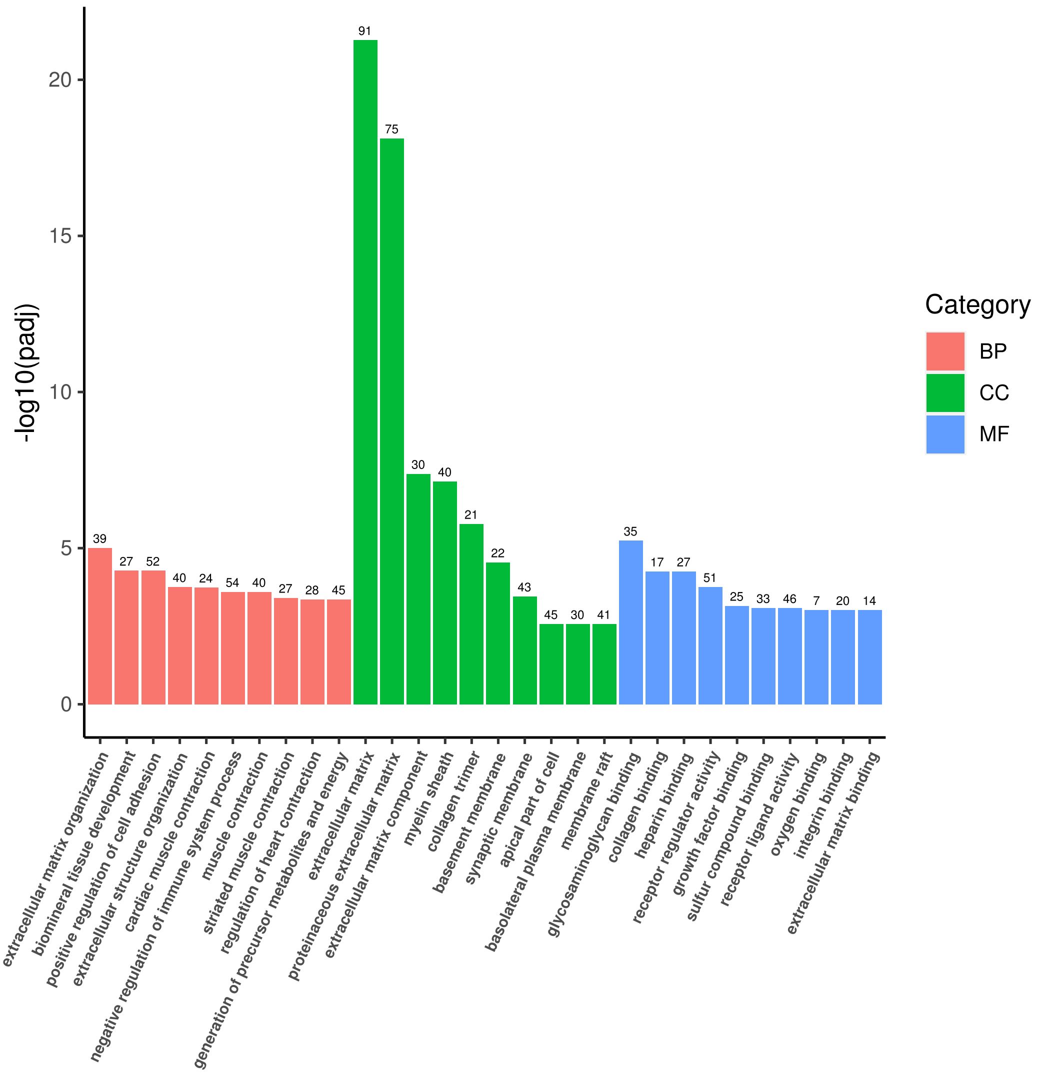

Supplement Fig 1. GO enrichment analysis showing the most significant 30 Terms upregulated in enamel organs from weight matched ELA as compared to control mice. Major categories include biological processes (BP), cell components (CC), and molecular functions (MF).

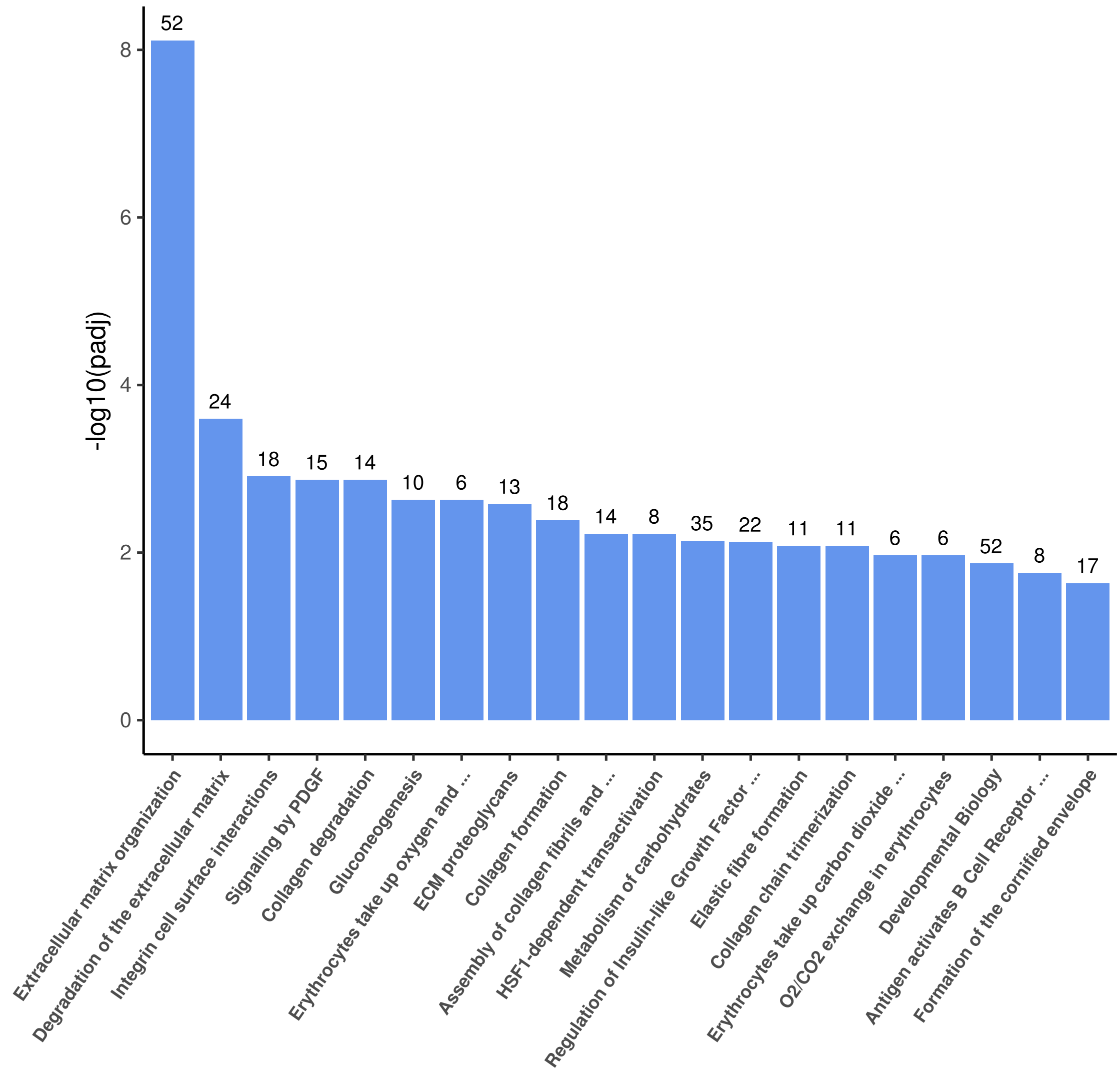

Supplement Fig 2. In the Reactome enrichment results, the most significant 20 pathways are shown. The abscissa is the Reactome pathway, and the ordinate is the significance level of the pathway enrichment. Higher values correspond to high significance.

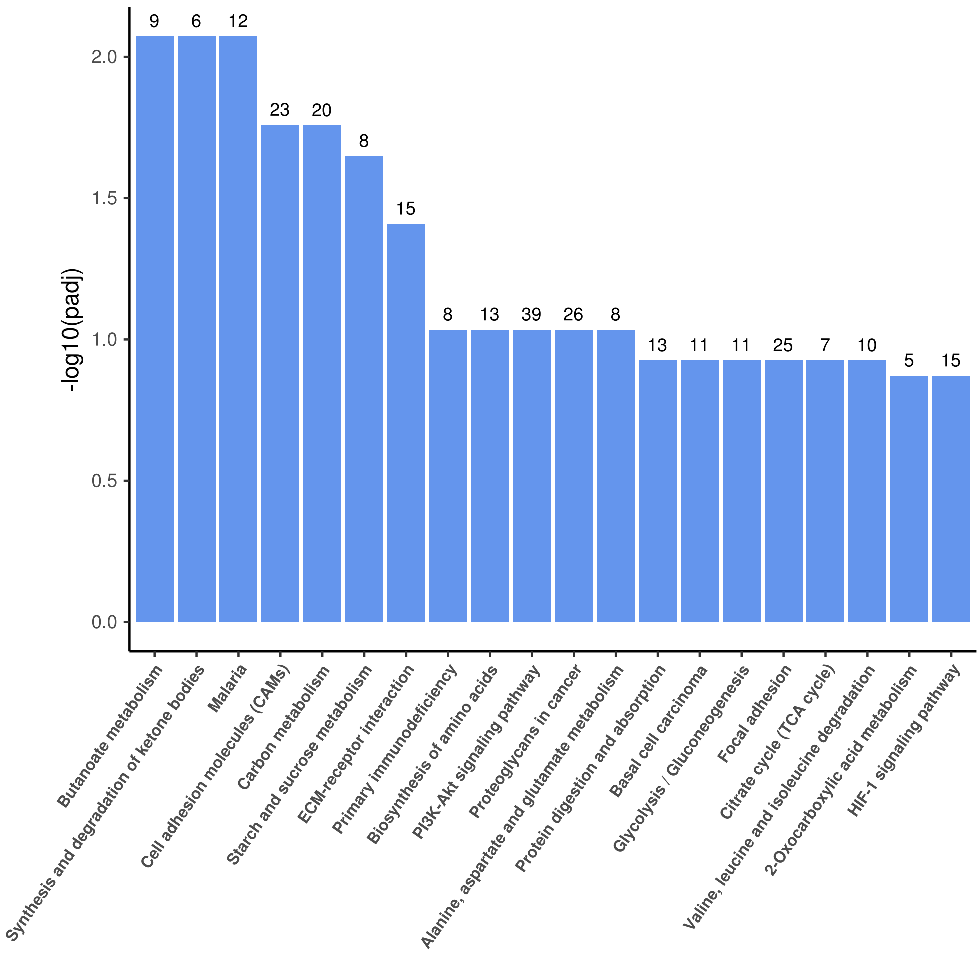

Supplement Fig 3) The most significant 20 KEGG pathways are shown. The abscissa is the KEGG pathway, and the ordinate is the significance level of the pathway enrichment. Higher values correspond to higher significance.
